# Predictive model for postoperative pneumonia in patients with esophageal cancer after esophagectomy

**DOI:** 10.3389/fonc.2025.1529308

**Published:** 2025-02-14

**Authors:** Jing Chen, Qian Xiang, Xiao-Jia Zheng, Xiao-yan Jiang

**Affiliations:** Department of Healthcare-associated Infection Control Center, Sichuan Provincial People’s Hospital, University of Electronic Science and Technology of China, Chengdu, China

**Keywords:** esophageal cancer, postoperative pneumonia, predictive model, esophagectomy, risk factor

## Abstract

**Background:**

Pneumonia is one of the most common complications after esophagectomy and a risk factor affecting postoperative survival of esophageal cancer. The aim of this study was to identify risk factors and construct a predictive model for postoperative pneumonia (POP) in esophageal cancer.

**Methods:**

This retrospective cohort study included esophageal cancer patients who underwent therapeutic esophagectomy from June 2019 to December 2023. Least absolute shrinkage and selection operator (LASSO) regression was used to screen predictive factors for POP, and a nomogram was constructed based on the selected predictive factors after screening. The performance of the model was evaluated using the area under the receiver operating characteristic curve (AUC), calibration curve, and decision curve analysis (DCA).

**Results:**

A total of 667 esophageal cancer patients who underwent esophagectomy were included, of whom 61 (9.1%) developed postoperative pneumonia. After LASSO regression analysis, factors independently associated with POP included mechanical ventilation for more than 2 days (P=0.000) and blood transfusion (P=0.003). A nomogram was constructed based on these independent risk factors. The AUC of the predictive model for POP was 0.839 (95%CI: 0.768-0.911). The internal verification result showed a good discriminative power and the DCA results demonstrated a good predictive value.

**Conclusion:**

The predictive model constructed in this study can predict the risk of POP in patients with esophageal cancer, and may promote early intervention for high-risk patients by clinicians to reduce the incidence of POP.

## Introduction

Esophageal cancer is a common malignant tumor, ranking eighth in the global incidence of diseases, and is also one of the most common causes of cancer-related deaths worldwide (1, 2). Esophagectomy is an important method for treating esophageal cancer. Postoperative pulmonary complications are the most common complication after esophagectomy, and pneumonia is the major pulmonary complication after esophagectomy, with an incidence rate between 20-40% (3–6). Postoperative pneumonia (POP) can lead to respiratory failure, prolonged hospitalization, increased hospitalization costs, and even tumor recurrence or death (7). In the perioperative management of esophageal cancer, prevention and control of POP are essential.

Esophageal cancer patients represent a unique population with distinct risk factors due to the invasive nature of esophagectomy, and associated respiratory challenges. The risk factors that can cause POP after esophagectomy included age, gender, obesity, and long operation time, and so on (8–10). Previous studies reported that POP was an independent risk factor affecting overall survival (11, 12). Therefore, identifying high-risk patients for postoperative pneumonia is of great significance for improving the prognosis of patients with esophageal cancer after esophagectomy. Currently, the risk factors for postoperative pneumonia in esophageal cancer varied among different studies. In addition, there are few predictive models for pneumonia after esophagectomy in patients with esophageal cancer. In this study, we aimed to analyze the risk factors of POP and construct a risk prediction model, in order to provide theoretical basis for optimizing prevention and control programs.

## Materials and methods

### Patients

A retrospective analysis was performed in Sichuan Academy of Medical Sciences and Sichuan People’s Hospital for the period June 2019 to December 2023. The study included patients who were histologically diagnosed with esophageal cancer and underwent radical surgery. The exclusion criteria were patients with incomplete clinical data. Patients who underwent endoscopic mucosal dissection to remove tumor and with incomplete clinical data were excluded. This study was approved by the Ethics Committee of Sichuan Academy of Medical Sciences and Sichuan People’s Hospital. Due to retrospective research and no negative impact on patients, the review committee waived the requirement for informed consent.

### Definition of postoperative pneumonia

Due to the lack of a unified diagnostic standard for POP, our hospital has developed a monitoring definition for POP based on active literature review and practical considerations (13–19). POP was defined as one or both lungs infection diagnosed within 30 days after surgery based on the following radiological criteria and clinical feature: (1) Chest X-rays or computed tomography (CT) scans showing new patchy infiltrates, consolidation of lobes/segments, ground glass opacities, interstitial changes or cavities, with or without pleural effusion; (2) respiratory symptoms, including coughing, purulent discharge, or difficulty breathing; (3) fever (body temperature ≥38°C) without other known causes or hypothermia (body temperature ≤36°C); (4) signs of lung consolidation, such as moist vales of lung pulmonary moist rales; (5) peripheral blood white blood cell (WBC) count ≥ 10 × 10^9^/L or WBC count ≤ 4 × 10^9^/L; (6) older adult individuals aged ≥70 years old with a change in mental status for no other apparent reason. Pneumonia was diagnosed if the imaging examination results were positive and the patient presented with any of the above clinical features.

### Data collection

Data collection was conducted through the hospital electronic medical record system and the hospital infection management system. Information of patients undergoing esophageal cancer surgery in our hospital were collected, including gender, age, underlying diseases, operation duration, intraoperative blood loss, mechanical ventilation, postoperative test results, etc. The data would be entered into an Excel spreadsheet and verified by two dedicated infection control personnel after collection. Patients would be divided into pneumonia group and no pneumonia group based on whether postoperative pneumonia occurred.

### Statistical analyses

Quantitative data are expressed as mean ± standard deviation or median (range), depending on whether it conforms to normal distribution. The student’s t-tests, χ^2^ test, or Mann-Whitney U test (as appropriate) was performed to analyze the differences between groups. We used the least absolute shrinkage and selection operator (LASSO) regression to screen the best predictive factors for POP, and construct a nomogram based on the screened factors. The performance of the nomogram was internally validated by the area under the receiver operating characteristic curve (AUC), calibration curve, and decision curve analysis (DCA). Statistical analysis of the data was performed by using R version 4.2.0 (https://www.r-project.org/). P < 0.05 was considered statistically significant.

## Results

### Patient characteristics

A total of 667 esophageal cancer patients who underwent esophagectomy were enrolled in this study. Among these patients, there were 561 males and 106 females, with a median age of 65 years (range 40-84 years). 61 patients developed POP, with an incidence rate of 9.1%. The length of hospital stay and hospitalization expenses in POP group were higher than those in the non-POP group (P<0.001). The postoperative in-hospital mortality rate of these patients was 2.8%, with a significantly higher mortality rate in the POP group compared to the non-POP group (P<0.001). The characteristics of the patients were summarized in **Table 1**.

**Table 1:** Patient characteristics in this study.

Variables	Total (n = 667)	Patients without POP (n = 606)	Patients with POP (n = 61)	P
Demographic characteristics				
gender, n (%)				0.005
male	561 (84.1)	502 (82.8)	59 (96.7)	
female	106 (15.9)	104 (17.2)	2 (3.3)	
age, median (IQR)	65.0 (57.0, 70.0)	64.0 (57.0, 70.0)	67.0 (61.0, 72.0)	0.024
Underlying disease				
hypertension, n (%)				0.065
0	540 (81)	496 (81.8)	44 (72.1)	
1	127 (19)	110 (18.2)	17 (27.9)	
COPD, n (%)				0.337
0	654 (98.1)	595 (98.2)	59 (96.7)	
1	13 (1.9)	11 (1.8)	2 (3.3)	
diabetes, n (%)				0.561
0	631 (94.6)	574 (94.7)	57 (93.4)	
1	36 (5.4)	32 (5.3)	4 (6.6)	
Chronic heart disease, n (%)				0.023
0	664 (99.6)	605 (99.8)	59 (96.7)	
1	3 (0.4)	1 (0.2)	2 (3.3)	
Laboratory indicators				
Preoperative ALB, mean ± SD	40.6 ± 3.6	40.6 ± 3.5	40.4 ± 4.0	0.702
preoperative HB, median (IQR)	134.0 (123.0, 145.0)	134.0 (123.0, 145.0)	134.0 (126.0, 147.0)	0.267
preoperative GLU, median (IQR)	5.1 (4.7, 5.7)	5.1 (4.7, 5.6)	5.0 (4.6, 5.7)	0.944
postoperative ALB, median (IQR)	38.9 (35.8, 42.6)	39.1 (35.9, 43.2)	37.6 (35.5, 40.4)	0.050
postoperative HB, median (IQR)	117.0 (97.0, 132.0)	118.0 (97.0, 132.0)	99.0 (97.0, 130.0)	0.268
postoperative GLU, median (IQR)	7.3 (6.0, 8.9)	7.1 (5.9, 8.6)	9.1 (8.4, 9.8)	< 0.001
surgical procedure				0.953
thoracotomy, n (%)	199 (29.8)	181 (29.9)	18 (29.5)	
thoracoscope, n (%)	468 (70.2)	425 (70.1)	43 (70.5)	
Inpatient management				
length of stay before surgery, median (IQR)	4.0 (3.0, 7.0)	4.5 (3.0, 7.0)	4.0 (3.0, 6.0)	0.425
blood transfusion, n (%)				< 0.001
0	521 (78.1)	491 (81)	30 (49.2)	
1	146 (21.9)	115 (19)	31 (50.8)	
mechanical ventilation for more than 2 days, n (%)				< 0.001
0	623 (93.4)	594 (98)	29 (47.5)	
1	44 (6.6)	12 (2)	32 (52.5)	
CVC, n (%)				0.023
0	113 (16.9)	109 (18)	4 (6.6)	
1	554 (83.1)	497 (82)	57 (93.4)	
indwelling time of CVC, median (IQR)	10.0 (7.0, 13.5)	10.0 (7.0, 13.0)	12.0 (8.0, 21.0)	< 0.001
Clinical outcome				
length of hospital stay, median (IQR)	17.0 (14.0, 22.0)	17.0 (14.0, 21.0)	21.0 (16.0, 28.0)	< 0.001
death, n (%)	19 (2.8)	6 (1)	13 (21.3)	< 0.001
hospital costs, median (IQR)	96175.0 (86082.2, 108719.6)	94803.7 (85623.5, 105637.1)	136013.0 (101468.0, 174828.0)	< 0.001

POP, postoperative pneumonia; COPD, chronic obstructive pulmonary disease; ALB, albumin; HB, hemoglobin; GLU, blood glucose; CVC, central venous catheterization

### Screening of predictive factors

**Figure 1:**
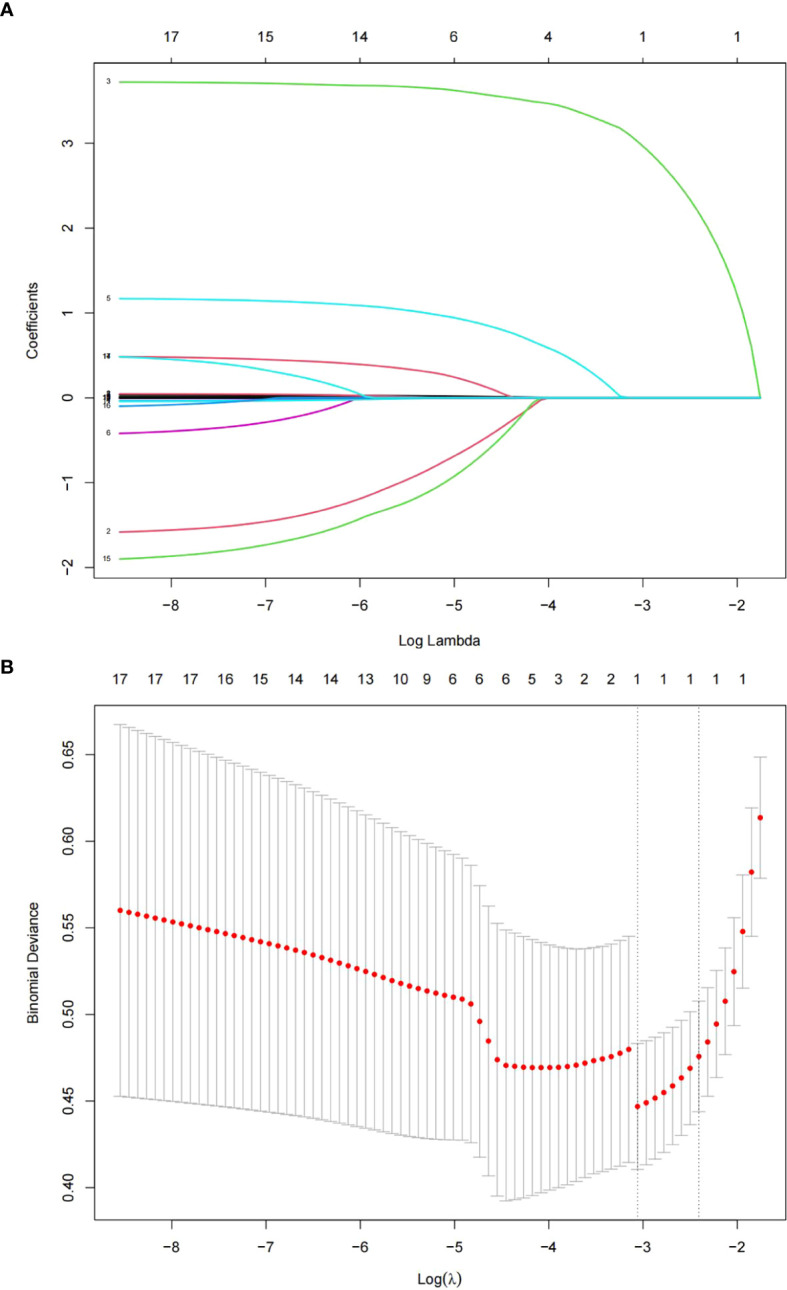
The LASSO regression method was used to screen predictive factors. **(A)** Changes in clinical related factors and penalty parameters (λ). **(B)** Based on cross validation and minimum criteria, 17 factors with penalty parameter (λ) in the model were adjusted.

After LASSO regression analysis, factors independently related to POP included mechanical ventilation for more than 2 days (P=0.000) and blood transfusion (P=0.003) (**Figures 1A, B** and **Table 2**). Through LASSO analysis, 2 potential predictive factors were initially selected (**Table 2**).

**Table 2:** Multivariate logistic regression analysis of the risk factors screened by LASSO regression.

Variable	P. value
mechanical ventilation for more than 2 days	0.000
blood transfusion	0.003

LASSO, least absolute shrinkage and selection operator.

### Construction of prediction model

**Figure 2:**
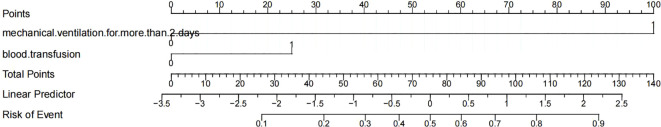
Nomogram for predicting postoperative pneumonia in in patients with esophageal cancer based on the independent risk factors.

**Figure 3:**
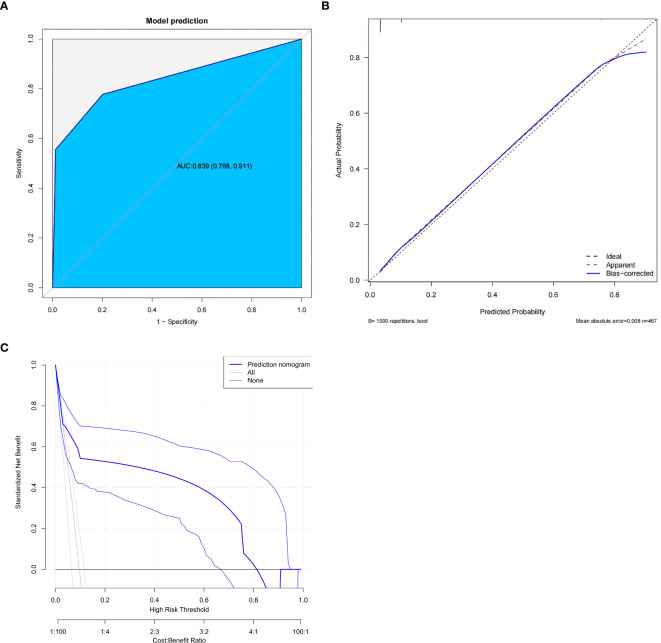
Evaluation of the predictive model. **(A)** Receiver operating characteristic (ROC) curve. **(B)** calibration plot. **(C)** decision curve analysis plot.

We use predictive factors to construct a POP prediction model for esophageal cancer patients, presented as a nomogram (**Figure 2**). In the nomogram, for each patient, each variable obtained a point from each variable axis, and the total number of points obtained was located on the total point axis, which reflected the probability of developing POP (20). The area under the ROC curve of this model was 0.839 (95%CI: 0.768-0.911) (**Figure 3A**), and the calibration curve indicated a good match between the actual observed values and the predicted values (**Figure 3B**). The predicted value of the calibration slope test was consistent with the actual result with the slope equal to 1. Decision curve analysis (DCA) indicated that the model had good predictive value (**Figure 3C**).

## Discussion

Surgery is the main treatment method for esophageal cancer, which can control the progression of the patient’s condition. POP is one of the most common complications after esophageal cancer radical surgery and a risk factor affecting postoperative survival of esophageal cancer (11). In this study, patients with POP had longer hospital stays, higher mortality rates, and higher hospitalization costs than those without POP. Therefore, the early implementation of preventive and control measures to reduce the incidence of POP has a positive effect on improving patient prognosis.

Our research indicated that mechanical ventilation for more than 2 days, blood transfusion were independent risk factors for POP. Patients with esophageal cancer who fail to meet the criteria for extubation postoperatively will continue respiratory support through mechanical ventilation. However, prolonged tracheal intubation would disrupt the normal barrier of the respiratory tract, increase the risk of bacterial infections and induce ventilator-associated pneumonia (21). At the same time, it can also weaken or disappear the patient’s cough reflex, causing respiratory secretions to be unable to be discharged autonomously and increasing the risk of VAP. With prolonged mechanical ventilation time, gastrointestinal contents are more prone to reflux, causing significant damage to airway mucosal tissue and leading to the translocation and colonization of opportunistic pathogens, resulting in lower respiratory tract infections. The duration of mechanical ventilation is closely related to the occurrence of pneumonia (22). Therefore, special attention should be paid to patients who are unable to have their tracheal intubation removed postoperatively or those whose condition worsen requiring mechanical ventilation. For these patients, postoperative pulmonary function rehabilitation should be to strengthened to facilitate early tracheal extubation.

Red blood cell (RBC) transfusion is necessary when there is substantial blood loss during surgery. Massive transfusion of allogeneic RBCs can cause transfusion-related immune regulation, and the storage time of RBCs may be the core of their immune regulatory effect (23). Previous studies found that soluble CD40 ligands accumulated in stored blood components and activated neutrophils through CD40, potentially affecting transfusion-related acute lung injury (24). This may explain the association between blood transfusion and increased risk of POP. A previous large-scale observational cohort study showed that intraoperative RBC transfusion was associated with increased risk of nosocomial infection after surgery in cardiac surgery patients (25). The optimal use of blood transfusion should be to provide sufficient blood transfusion to maximize clinical outcomes while avoiding unnecessary exposure to infection risks (26). Therefore, strengthening perioperative blood transfusion management can contribute to reducing the risk of POP.

Nomograms has become a valuable tool in clinical practice, which can help predict the probability of event occurrence or survival (27). In the present research, the POP prediction model showed a good discrimination and calibration, which had significant guidance for reducing the occurrence of pneumonia following esophagectomy. Therefore, we can use this model to predict the probability of POP occurrence in patients after esophagectomy based on risk factors and intervene in advance to reduce the incidence of POP.

In this study, we developed a monitoring definition for POP in our hospital based on relevant guidelines, expert consensus, and multiple literature, and diagnosed 9.1% of POP in esophageal cancer patients. However, in previous studies, there had been widespread differences in the incidence of POP. Although the patient’s baseline condition, surgical procedure, and postoperative care may affect the incidence of POP, the lack of a standardized definition for POP may result in some POP not being correctly identified, leading to such differences. The current research has not reached a consensus on the definition of POP (5, 28), and further research is needed to clarify a unified definition of POP.

There were several potential limitations in our study. Firstly, this study was a retrospective single-center study conducted in a single large tertiary hospital, not multicenter research. Multi-center studies with more diverse populations would be needed to confirm the external validity of our results. Secondly, the number of patients requiring mechanical ventilation for more than 2 days in this study is relatively small, which may affect the statistical power. Therefore, it is necessary to further validate in larger cohorts and explore the optimal cut-off value for mechanical ventilation duration. Thirdly, due to the limited sample size, our predictive model has only been validated internally. External validation in a larger cohort, ideally through multicenter collaboration, is essential for assessing the model’s robustness and applicability across different clinical settings. In the future, we will conduct large-scale multi-center research to further improve our model and enhance its predictive accuracy.

## Conclusion

In conclusion, we demonstrated that mechanical ventilation for more than 2 days and blood transfusion were important predictive factors for POP in esophageal cancer patients undergoing esophagectomy. Based on our nomogram, the occurrence of POP can be predicted, and preventive measures can be taken as early as possible to reduce the incidence of POP.

## Data Availability

The original contributions presented in the study are included in the article/supplementary material. Further inquiries can be directed to the corresponding author.
